# Distinguishing Benign and Malignant Findings on [^68^ Ga]-FAPI PET/CT Based on Quantitative SUV Measurements

**DOI:** 10.1007/s11307-022-01759-5

**Published:** 2022-08-23

**Authors:** M. Dabir, E. Novruzov, K. Mattes-György, M. Beu, K. Dendl, C. Antke, S. A. Koerber, M. Röhrich, C. Kratochwil, J. Debus, U. Haberkorn, F. L. Giesel

**Affiliations:** 1 Department of Nuclear Medicine, Medical Faculty, Heinrich-Heine-University, University Hospital Dusseldorf, Moorenstrasse 5, 40225 Düsseldorf, Germany; 2 Department of Nuclear Medicine, University Hospital Heidelberg, Heidelberg, Germany; 3 Department of Radiation Oncology, Heidelberg University Hospital, Heidelberg, Germany

**Keywords:** FAPI PET, Equivocal/incidental finding, Accuracy, Diagnostic performance, Benign lesion

## Abstract

**Aim/Purpose:**

Fibroblast activation protein (FAP) is overexpressed by cancer-associated fibroblasts. However, activated fibroblasts have been shown to play a significant role also in certain benign conditions such as wound healing or chronic inflammation. Therefore, the current study aimed to identify whether FAPI uptake might differ between malignant lesions and benign conditions.

**Material and Methods:**

We retrospectively analyzed 155 patients with various cancer types who received [^68^ Ga]-FAPI-04/02-PET/CT between July 2017 and March 2020. SUV_max_, SUV_mean_, and lesion-to-background ratios (LBR) of FAPI uptake were measured in benign processes compared to malignant lesions (primary and/or 2 exemplary metastases). In addition, receiver operating characteristic (ROC) curve analysis was conducted to compare the predictive capabilities of semiquantitative PET/CT parameters. Furthermore, the sensitivity, specificity, optimal cutoff value, and 95% confidence interval (CI) were determined for each parameter.

**Results:**

Benign lesions exhibited significantly lower FAPI uptake compared to malignant lesions (mean SUV_max_ benign vs. malignant: 4.2 vs. 10.6; *p* < 0.001). In ROC analysis, cutoff values of these lesions (benign vs. malignant) were established based on SUV_max_, SUV_mean_, and LBR. The SUV_max_ cutoff value for all lesions was 5.5 and the corresponding sensitivity, specificity, accuracy, and AUC were 78.8%, 85.1%, 82.0%, and 0.89%, respectively.

**Conclusion:**

Our aim was to systematically analyze the pattern of FAPI uptake in benign and malignant processes. This investigation demonstrates that FAPI uptake might be useful to differentiate malignant and benign findings due to different patho-physiological origins.

**Supplementary Information:**

The online version contains supplementary material available at 10.1007/s11307-022-01759-5.

## Introduction

Recently, novel and highly promising PET tracers targeting fibroblast activation protein (FAP) by small molecule FAP inhibitors (FAPIs) have been introduced [1]. [^68^ Ga]-FAPI offers an additional pan-tumor tracer with excellent diagnostic performance and theranostic potential to the current standard oncological tracer 2-deoxy-2-[18F]fluoro-d-glucose ([^18^F]-FDG). One of the advantages of this new approach represents its independence from glucose metabolism and the overexpression of FAP in more than 90% of human epithelial cancer cells [1]. Concurrently, FAP ligands demonstrate a rather low uptake in non-malignant tissue, ensuring favorable tumor-to background ratios (TBR) in malignant lesions. Furthermore, sharp image contrasts can be achieved [2], particularly in liver, brain, and oral mucosa, which are characterized by moderate to high physiological FDG uptake. Therefore, novel diagnostic opportunities such as FAPI imaging are crucial to overcome challenges in these tumor entities that display unfavorable TBR with FDG PET/CT (i.e., hepatic or pancreatic cancers) [3].

FAP is a type II serine protease belonging to the dipeptidyl peptidase 4 family and has both post-proline dipeptidyl peptidase and endopeptidase activity [4, 5]. FAP is one of the surface markers expressed by cancer-associated fibroblasts (CAFs), which are part of the stroma in various malignant tumors [6]. The tumor stroma consists of endothelial cells, the basement membrane, extracellular matrix, and immune cells. In epithelial malignancies such as breast, colon, and pancreatic cancer, CAFs account for about 90% of the tumor mass [7]. CAFs secrete a number of molecules, mostly growth factors and cytokines (a prominent one being the transforming growth factor β (TGFβ)), which are shown to promote epithelial to mesenchymal transition [8]. Thus, FAP overexpression is associated with tumor immunomodulation leading to increased local tumor invasion, metastasis, and overall poor prognosis [6].

However, FAP is also selectively expressed in certain benign conditions and in normal tissues such as during wound healing [9], tissue remodeling [10], fibrosis, at sites of inflammation like arthritis [11, 12], in atherosclerotic plaques [13], and after myocardial infarction [14]. Moreover, in immunohistochemical staining, elevated FAP levels are also present in the cervix and uterine stroma (especially during the proliferative cycle) as well as in the pancreas [15].

Thus, the distinction of incidental or confounding equivocal findings in the clinical setting appear to be one of the most frequent pitfalls. Although some data has already been published with respect to characterization of FAPI findings in benign tissues [16–18], predictive criteria for further clarification of such findings have not been described in the literature, yet. The aim of this work is to define or develop semiquantitative parameters that allow accurate prediction between benign and malignant FAPI uptake.

## Methods

### Patients

In this study, we retrospectively analyzed 155 oncological patients aged 31 to 93 with various malignancies (Table 1). Patients were referred by their attending oncologist or radiation oncologist and the initial evaluation of the data was conducted in a clinical setting at the University Hospital Heidelberg. The further statistical analysis was performed at the University Hospital Dusseldorf. Electronic patient records were additionally selectively analyzed to gather further information on clinical diagnoses. Several patients of this cohort were already evaluated regarding their malignancies under a different scope [2, 19–22], as well as their benign uptake in hormone-responsive organs including endometrium, breast, and ovary [23].

**Table 1:** Primary tumors detected on [^68^ Ga]-FAPI PET/CT

Tumor entity	Number of patients	Mean SUV_max_	Range SUV_max_
Pancreatic cancer	32	11.55	4.9–19.39
Head and neck cancer	27	12.16	2.88–21.59
Colorectal cancer	18	9.42	5.69–13.97
Lung cancer	11	13.5	3.64–19.3
Gastroesophageal cancer	10	13.21	4.8–23.9
Hepatobiliary cancer	9	12.97	4.5–17.6
Prostate cancer	9	8.79	3.46–14.6
Thyroid cancer	8	5.8	2.3–13.7
Breast cancer	7	12.17	7.4–27.32
CUP	6	15.55	3.35–21.8
Anal cancer	3	19.8	13.2–26.4
Small intestine cancer	2	10.17	NA
Cervical cancer	2	11.73	NA
Endometrial cancer	1	7.8	NA
Other cancers	10	7.8	3.6–14.7
Total	155		

All patients gave written informed consent before undergoing [^68^ Ga]-FAPI PET/CT on an individual patient basis following national regulations, the Declaration of Helsinki and Good Clinical Practice (GCP). The radiopharmaceutical was synthesized and labeled under the terms of the German Pharmaceutical Act §13(2b). The retrospective evaluation of data was approved by the ethics committee of Heidelberg University (S-358/2022).  

### Radiopharmaceuticals and [^68^ Ga]-FAPI PET/CT Imaging

PET scans were performed in 3D mode with an acquisition time of 3–5 min/bed position (Supplement Table 1). Two FAP ligands with similar chemical compositions were used: FAPI-02 (*n* = 24) and FAPI-04 (*n* = 131). Radiosynthesis and labeling of ^68^ Ga-labeled FAP ligands were performed as described in previous publications [8, 24]. Median injected activity was 239 MBq (range 118–340). Imaging data was acquired 1 h (*n* = 155) after tracer application. Whole body images, including the patients head to mid thighs, were acquired.

### Image Analysis

[^68^ Ga]-FAPI uptake in tumor lesions and incidental/equivocal findings was quantified by mean and maximum standardized uptake values (SUV_mean_ and SUV_max_). Lesion-to-background ratio (LBR) was calculated by dividing the SUV_max_ of the lesions (both benign and malignant) by SUV_mean_ of blood pool to quantify the imaging contrast.

Circular regions of interest (ROI) were placed by one UHD investigator (MD; supervised by FLG) on axial slices around lesions with focally increased tracer uptake in organs and tissues not affected by primary tumor or metastasis. The ROIs were then automatically incorporated into a 3-dimensional volume of interest (VOI) with e.soft software (Siemens) using a 60%-isocontour. The [^68^ Ga]-FAPI PET/CT scans were previously analyzed in consensus by a board-certified radiologist, a board-certified radiation oncologist, and two board-certified nuclear medicine physicians.

Moderate to high organ or tissue tracer uptake and biodistribution were quantified by mean and maximum standardized uptake values (SUV_mean_ and SUV_max_). For comparison to benign FAPI uptake, SUV_max_ and SUV_mean_ of the primary (if present), blood pool and two representative metastatic lesions (if present) were measured as well. Evaluation of moderate to high FAPI uptake in organs and tissues not affected by malignancy was conducted with a 1-cm diameter (for the small organs and tissues like thyroid, esophagus, arteries, myocardium, oral mucosa, ovary, prostate) or 2-cm diameter (liver, pancreas, aortic lumen content, lung, mamma, endometrium, joints, wounds, intestines) sphere placed inside the organ parenchyma or tissue.

Primary lesions and metastases were defined considering imaging-based diagnosis, clinical diagnosis, or histopathological confirmation. Similarly, for the diagnosis of benign FAPI uptake, PET/CT imaging, data from other imaging modalities, medical history, and results of histological biopsy were taken into consideration. For further evaluation, tracer uptake of incidental or equivocal findings, blood pool, and tumor lesions were quantified by SUV_max_ and SUV_mean_.

Regarding FAPI uptake, the lesions were categorized as low (SUV_max_ < 1.5), moderate (SUV_max_ 1.5–4) and high (SUV_max_ > 4); the lesions with low uptake were excluded from statistical analysis and further evaluation.

### Statistics

We used descriptive analyses for demographics, tumor characteristics, and tracer uptake. The descriptive analysis of the tracer uptake by SUV_max_, SUV_mean_, and LBR values between tumor and benign lesions were displayed using box plots (Fig. 2). Comparison of FAPI uptake between malignant and benign lesions was performed using the Mann–Whitney *U* test. A *p* value of < 0.05 was considered statistically significant. The descriptive statistical analyses were performed using Excel for Mac Version 15.41 (Microsoft) and SigmaPlot 11.0 (Systat Software Inc.).

Receiver operating characteristic (ROC) curve analysis and the area under the ROC curve (AUC) were used to compare the predictive capabilities of semiquantitative PET/CT parameters regarding the differential diagnosis of equivocal findings and the sensitivity, specificity, optimal cutoff value, and 95% confidence interval (CI) were also calculated for each parameter. This statistical analysis was performed using MedCalc® Statistical Software version 20.011 (MedCalc Software). Optimal cutoffs were defined by Youden’s index as those resulting in high sensitivity corresponding to highest negative predictive value or the maximum specificity for a given minimum level of sensitivity. The comparison of different AUCs was conducted by the method described by DeLong et al. [25]

## Results

### Study Population

The cohort consists of 155 patients (70 females and 85 males) with a median age of 67 years (range 31–93 years) who underwent [^68^ Ga]-FAPI PET/CT scans between July 2017 and March 2020. Fifty different types of malignancies were included (Table 1). A total of 292 malignant lesion were evaluated, of which 82 were primary tumor lesions and 210 were metastatic lesions. The following cancer types were included: head and neck cancer (*n* = 27), lung carcinoma (*n* = 11), hepatobiliary cancer (*n* = 9), pancreatic cancer (*n* = 32), gastrointestinal tract cancer (*n* = 33), gynecologic cancer (*n* = 10), CUP (cancer of unknown primary, *n* = 6), prostate cancer (*n* = 9), and miscellaneous malignancies (*n* = 10) such as desmoid cancer, insulinoma, leiomyosarcoma, pheochromocytoma, thymic carcinoma and pancreatoblastoma.

###  [^68^Ga]-FAPI uptake in malignant and benign lesions (Benign vs. Malignant)

**Fig. 1:**
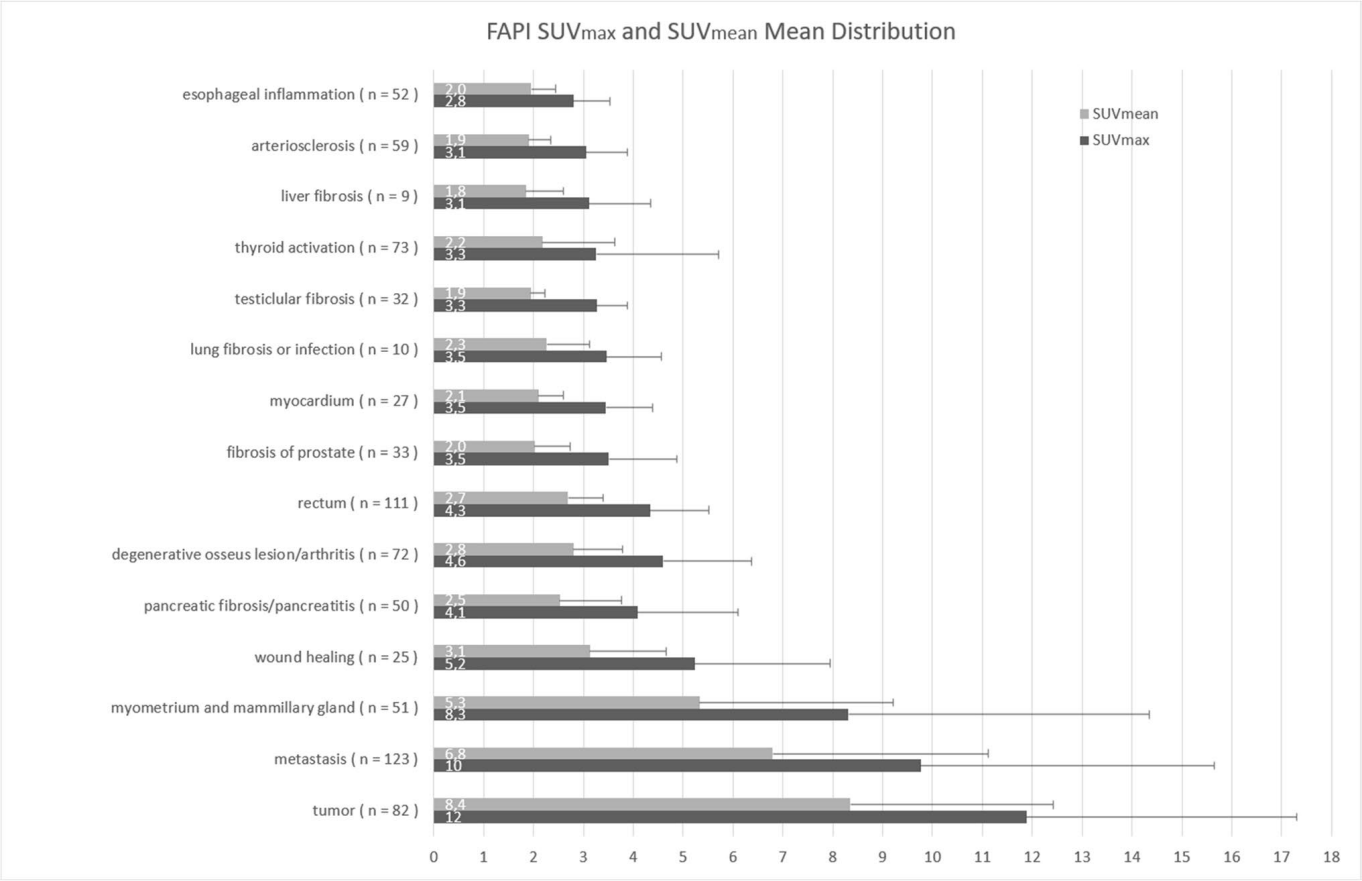
Mean distribution (SUV_max_ and SUV_mean_) of [^68^ Ga]-FAPI in organs not affected by metastases/benign lesions and in malignant lesions (mean values and standard deviation).

Distribution of [^68^ Ga]-FAPI in benign and malignant lesions (primary tumor and metastases) is presented in Fig. 1. The mean SUV_max_ and median SUV_max_ of the primary tumors were 12.1 and 11.5 (range 2.9–27.8), respectively. The mean SUV_max_ and median SUV_max_ of all metastases were 10.0 and 8.4 (range 2.4–44.3), respectively. Regarding the overall 618 benign lesions in 155 patients, FAPI showed a significantly lower uptake compared to malignant lesions (mean SUV_max_ benign vs. malignant, 4.2 vs. 10.6; *p* < 0.001). Among the benign lesions, the uterine tissue (in particular, myometrium) demonstrated the highest FAPI uptake (*n* = 40, mean SUVmax: 9.9; *p* = 0.274) and represents the most outliers in the comparative statistical evaluation between benign and malignant lesions.

**Fig. 2:**
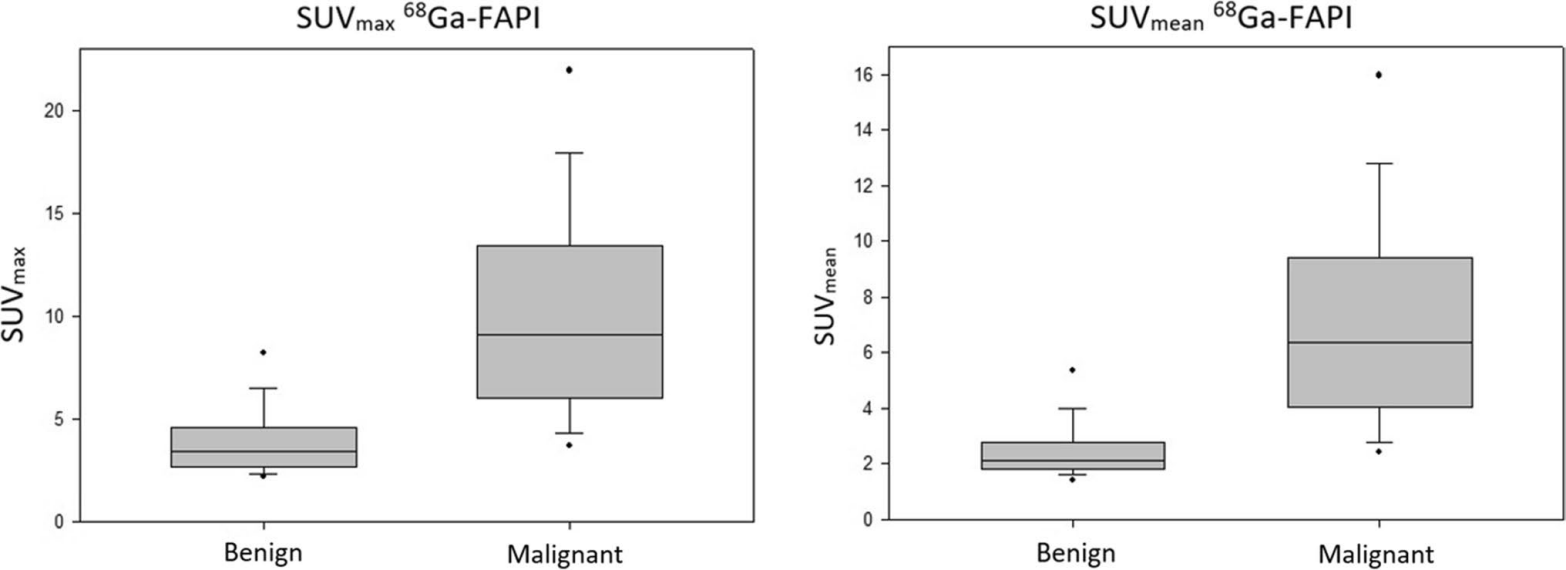
The boxplot analysis shows biodistribution (SUV_max_ and SUV_mean_) of [^68^ Ga]-FAPI regarding benign vs. malignant lesions (mean values and standard deviation).

**Fig. 3:**
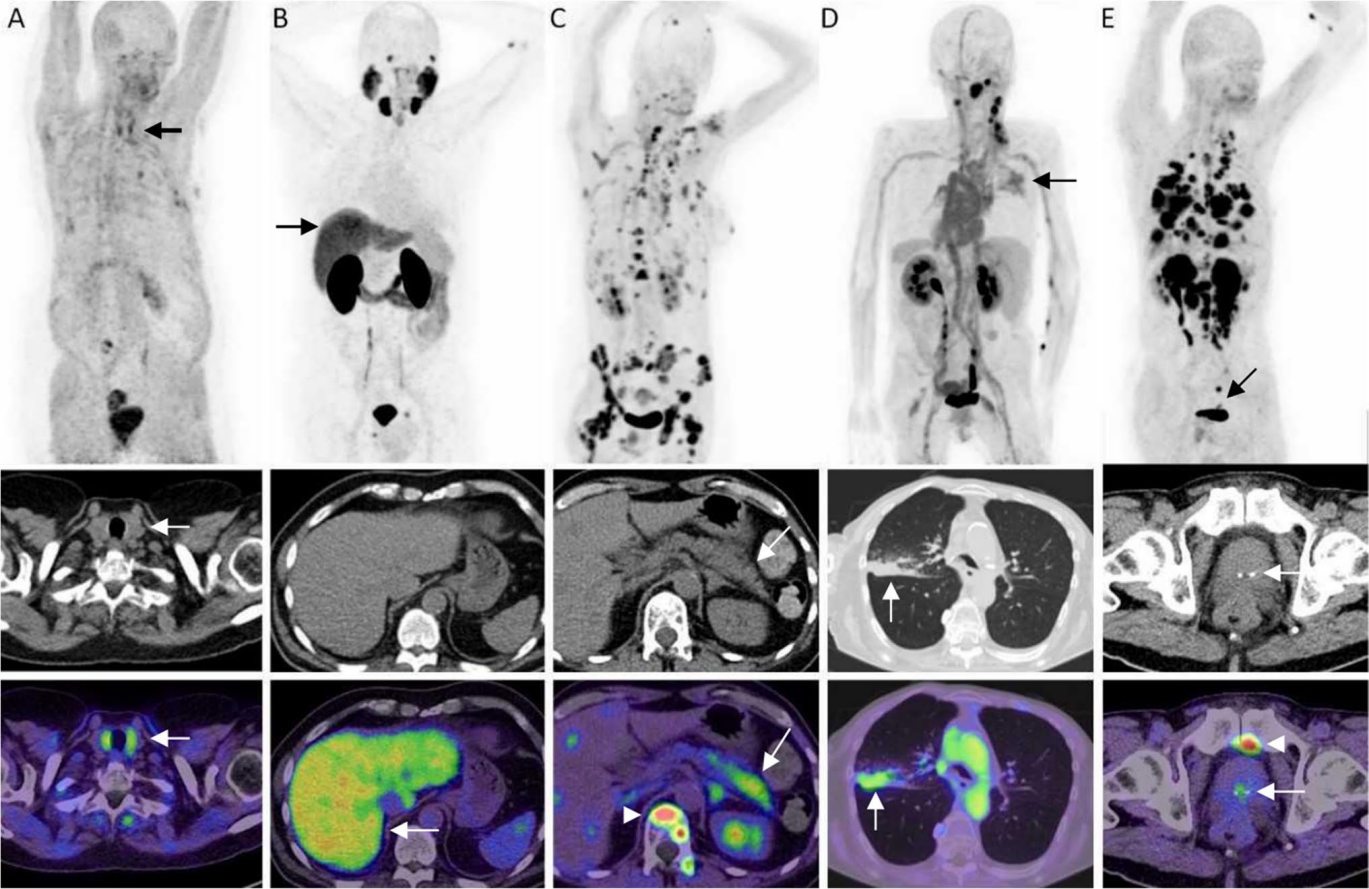
**A** A female 69-year-old patient with adenoid cystic carcinoma of the parotid gland showing a homogenous, high FAPI uptake of the thyroid with known Hashimoto thyroiditis (black arrow on MIP). **B** A 64-year-old male patient with esophageal cancer showing a diffuse, high uptake of FAPI of the whole liver (black arrow on MIP), while CT scan describes a nodular surface of the liver, concordant with fibrosis of the liver. A CT scan from January 2011 already described hepatic steatosis in this patient. **C** A 57-year-old patient with breast cancer showing a diffuse high uptake of FAPI in the corpus and tail of the pancreas (not shown on MIP, white arrow in transversal fusion image). A lab work performed 3 months after [^68^ Ga]-FAPI PET/CT scan showed slight elevated levels of LDH, potentially suggesting pancreatitis. Nonetheless, this differential diagnosis was not confirmed in further patient history. White arrowhead points to an osseous metastasis. **D** A 65-year-old patient with squamous cell carcinoma of the tongue and known chronic obstructive pulmonary disease showed an inflammatory pulmonary consolidation with an intense uptake of FAPI, which was deemed as pulmonary consolidation of the right upper lobe (black arrow on MIP, white arrow on transversal fusion image). **E** A 70-year-old patient with esophageal cancer showed a moderate uptake of FAPI in the prostate lobe (black arrow on MIP, white arrow on transversal fusion image) specifically in sight of a focal calcification of the prostate. A CT scan performed 1 month prior described a benign prostate hyperplasia (white arrowhead points to osseous metastasis in the pelvis).

**Fig. 4:**
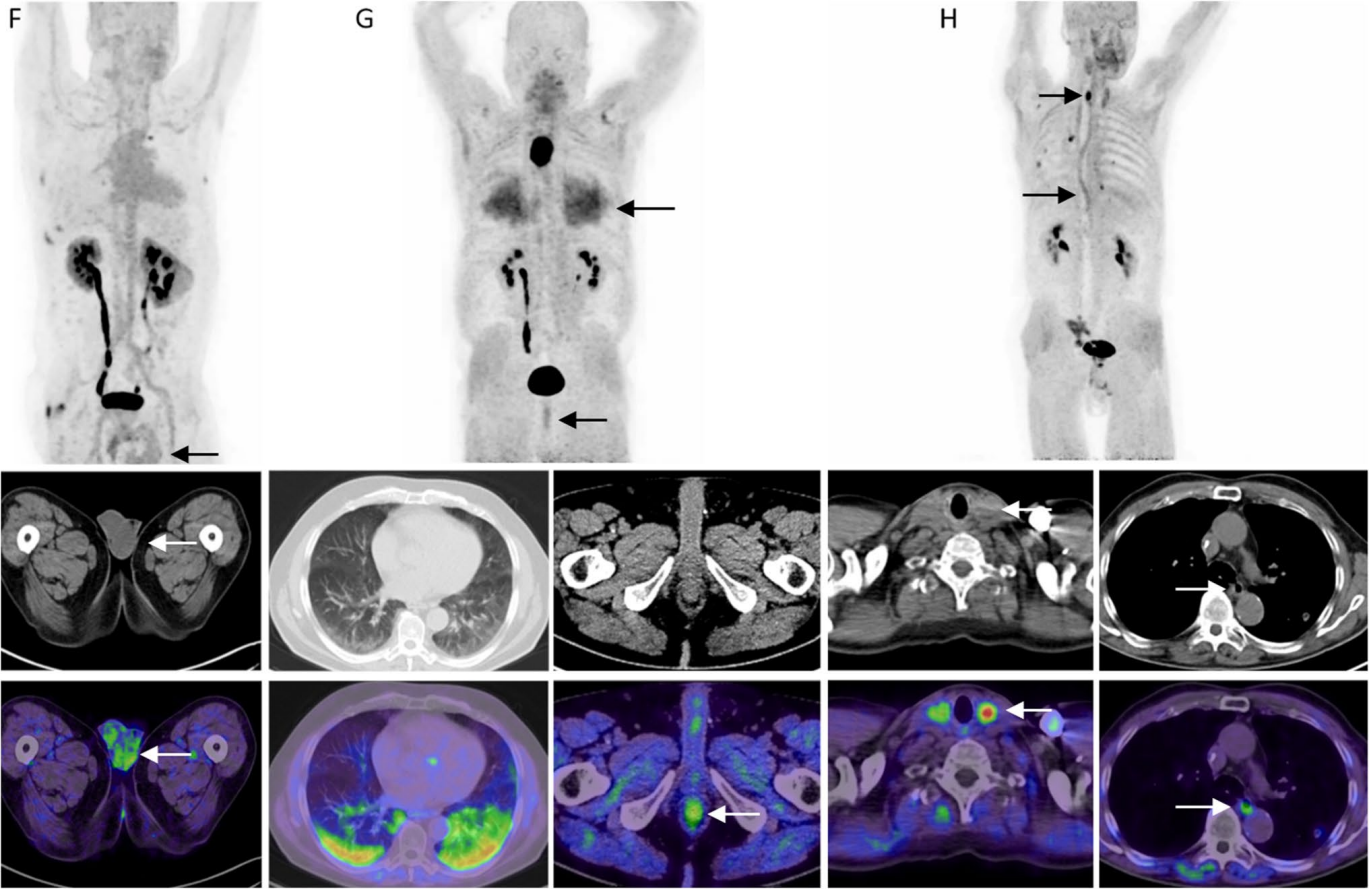
**F** A 60-year-old man with renal cell carcinoma showed a moderate, diffuse uptake of the testicles. As further side findings, BPH was noted. **G** A 76-year-old patient with esophageal cancer showing a diffuse, bilateral, high uptake of FAPI in the lungs, predominantly in the inferior lobe, fibrosis of the lung was diagnosed in the CT scan. Furthermore, this patient also showed moderate, diffuse uptake in the anal canal. No discernible pathologies were noted in the rectum or anal canal. **H** A 68-year-old-patient with colon cancer and two incidental findings; an intense uptake in the left lobe of the thyroid was noted. TSH levels were normal, and scintigraphy was recommended. Furthermore, the patient showed a moderate uptake in the middle and lower third of the esophagus without discernible esophageal pathologies in the CT scan or medical history.

However, in comparison to malignant lesions, the following benign lesions exhibited significantly lower tracer uptake. We observed moderate to high FAPI uptake at sites of wound healing/anastomosis (*n* = 25 mean SUV_max_ 5.2; *p* ≤ 0.001). In 50 cases, the pancreas demonstrated primarily low to moderate tracer uptake. Nevertheless, in ten cases, we observed slightly increased, diffuse tracer uptake most likely due to tumor-induced pancreatitis or postoperative pancreatitis after partial pancreatectomy (mean SUV_max_: 4.1; *p* < 0.001). Diffuse or focal uptake in the prostate was present in 33 patients (mean SUV_max_ 3.5; *p* ≤ 0.001). Other benign findings include the thyroid (*n* = 73 mean SUV_max_ 3.3; *p* ≤ 0,001), rectum/anal canal (*n* = 111, mean SUV_max_ 4.3; *p* < 0.001), testicles (*n* = 32, mean SUV_max_ 3.3; *p* < 0.001), sites of degenerative disease/arthritis (*n* = 72, mean SUV_max_ 4.6; *p* < 0.001), longitudinal in the esophagus (*n* = 52, SUV_max_ mean 2.8; *p* < 0.001), lung (*n* = 10, mean SUV_max_ 3.5; *p* = 0.001), atherosclerotic plaques (*n* = 59, mean SUV_max_ 3.1; *p* < 0.001), in the myocardium (*n* = 27, mean SUV_max_ 3.5, *p* < 0.001), and in the liver parenchyma (*n* = 9, mean SUV_max_ 3.1; *p* < 0.001) (Figs. 1, 2, 3, and 4).

### Accuracy of [^68^ Ga]-FAPI PET/CT

**Fig. 5:**
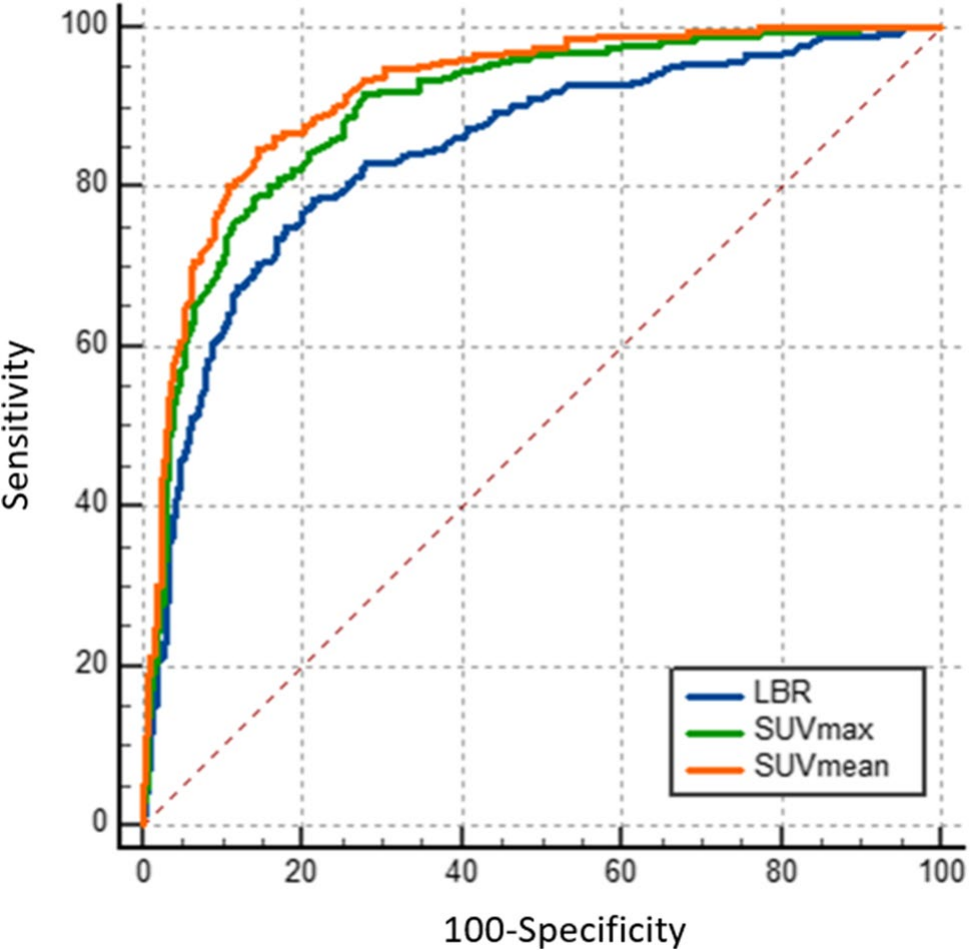
In the receiver operating characteristic (ROC) curve comparison, most favorable prediction rates (AUC > 90%) is seen in SUV_mean_

The optimal cutoff values for accurate determination of the lesions (benign vs. malignant) were established based on SUV_max_, SUV_mean_, and LBR. The SUV_max_ cutoff value for all lesions was 5.5 and the corresponding sensitivity, specificity, accuracy and AUC were 78.8%, 85.8%, 83,5% and 0.89, respectively (95% CI 0,876 - 0,916). The SUV_mean_ cutoff value for overall lesions was 3.3 and the corresponding sensitivity, specificity, accuracy and AUC were 84.9%, 85.3%, 85,2% and 0.91, respectively (95% CI 0,898 - 0,935). The LBR cutoff value for overall lesions was 4,1 and the corresponding sensitivity, specificity, accuracy and AUC were 75,0%, 82,06%, 79,78% and 0,84, respectively (95% CI 0,82 - 0,866). Comparisons of ROC curves showed AUC to be 89,7% for SUV_max_, 91,8% for SUV_mean_, and 84,3% for LBR, showing good prediction rates for SUV_max_ and SUV_mean_. The comparison of ROC curves represented the most favorable results for the SUV_mean_, even though all three parameters convince with good diagnostic accuracy (Fig. 5). Concerning malignant with a prevalence of 32,27% in this sample, the negative predictive value (NPV) and positive predictive value (PPV) with respect to SUV_max_ could be stated as 89.5% and 72.6%. In concordance with SUV_mean_ and LBR, the NPV and PPV would be stated as 92.2%, 73.4% and 87.32%, 66.58%, respectively.

## Discussion

This retrospective study primarily aimed to provide an overview of the frequency and clinical significance of incidental benign findings in [^68^ Ga]-FAPI PET/CT. Additionally, we sought to determine a reliable, accurate parameter or assessment method to facilitate imaging-based differentiation of those lesions. To this end, we analyzed a large patient cohort with a broad spectrum of histopathological verified cancer entities accompanied by confounding equivocal findings.

To distinguish between FAP expression of CAFs and the reactivation of the so-called quiescent fibroblasts in benign remodeling processes, i.e., wound healing and fibrotic changes, or physiological changes in hormone sensitive organs such as breast or uterus [26, 27] is still a clinical challenge.

Studies with smooth muscle cells and formation of atherosclerotic plaques revealed a strong correlation between an increase of FAP expression and atherosclerotic changes in arteries which seems to be a poor prognostic factor because of the formation of thin-cap human coronary plaques [26]. In our evaluation, we observed 59 cases with moderately elevated FAPI uptake not only at sites of visible atherosclerotic plaques, but also in the thoracic and abdominal arteries as well as coronary arteries that showed no detectable signs of arteriosclerosis. Presumably, the macrophage infiltration due to a subclinical atherogenesis leads to an increased FAP expression by TNFα-secretion [28]. However, this hypothesis requires further research to determine whether a decent increase of FAPI uptake within the vessel walls is a suitable predictive/prognostic factor for the atherosclerotic plaque formation and development of clinical manifestations.

As for FAPI uptake of the testicles, it has been established that seminiferous tubules are surrounded by the lamina propria, which consists of the basement membrane and the outer layers of myoid cells, connective tissue, and fibroblasts, whereas the outmost layers consist of mainly fibroblasts [29]. The composition of the peritubular wall can undergo dramatic changes. A study suggested that tryptase produced by peritubular mast cells may be directly involved in the development fibrosis, in particular the fibrotic thickening of the walls of the seminiferous tubules, causing infertility [30]. It might be possible, considering the advanced age of most male patients included in our study, that moderate FAPI uptake in the testicles might be associated with the these changes.

Among the benign lesions shown on [^68^ Ga]-FAPI PET/CT, we observed 50 cases with moderate to diffusely high FAPI uptake in the pancreas. Some of these patients have had a prior cancer surgery, e.g., partial resection or postoperative pancreatitis as surgical complication. In other cases, without a surgical history and no clinical information suggesting acute pancreatitis, this appears to be related to chronic pancreatitis or fibrotic changes within the parenchyma. Furthermore, 25 patients were identified demonstrating moderate FAPI uptake at the site of active wound healing after operation (i.e., laparotomy). Overall, the diagnosis of an early tumor recurrence at sites of wound healing might be challenging due to high background activity of the target organ during the early postoperative period.

Several studies report an upregulation of FAPI in patients with thyroiditis and fibrotic changes in the thyroid parenchyma whereas FAPI uptake in differentiated thyroid cancers remains low [19, 21]. We had a confirmed case of Hashimoto’s thyroiditis in a patient with a diffuse increased FAPI uptake. Yet, 75 patients without history of thyroid pathologies demonstrated a diffuse or focal, moderate FAPI uptake in the thyroid parenchyma. Diffuse uptake of the thyroid might be linked to fibrotic changes of the thyroid architecture (e.g., lymphocyte and fibroblast infiltration) due to undiagnosed autoimmune diseases, like Hashimoto thyroiditis [31], Graves’ disease [32], or immune-related thyroiditis caused by immunosuppressive therapy [33]. A recently published study supports this assumption [16].

Similarly, in 33 cases or 42% of male patients of our cohort, we noticed diffuse moderate uptake of FAPI in the prostate. The prevalence of prostatitis is estimated to range from 2.2 to 9.7% [34]; hence, a fourfold increase of incidental prostatitis seems very unlikely. Moreover, FAP expression in the benign prostate is shown to be relatively low. However, several studies report a crucial role of inflammation for the development and progression of benign prostatic hyperplasia (BPH) which has a high prevalence of more than 50% after 6th decade [35]. Hesterberg et al. demonstrated that FAP expression is significantly increased in the tumor microenvironment adjacent to Gleason grade 4 prostate cancer compared to benign prostate. Therefore, even a moderate, focal uptake of FAPI in the prostate should initiate diagnostic work-up in order to rule out prostate cancer [36] and an even distributed moderate to high FAPI uptake in the prostate might be assigned to BPH.

In concordance with previously conducted studies, we also noted a high, diffuse FAPI uptake in patients with fibrotic lung (12 cases) and liver diseases (9 cases). The application of FAPI for the diagnosis of primary or secondary hepatic malignancy is expected to be a promising diagnostic tool because of very low background uptake in liver tissue and thus excellent target-to-background ratio. However, the primary hepatic malignancies usually emerge in patients with fibrotic changes or advanced cirrhosis, which still might act as a limiting factor [37]. Moreover, intrahepatic expression of FAP seems to correlate with the degree of fibrosis [38]. Our evaluation seems to support these findings, as Fig. 3 shows a patient with liver cirrhosis demonstrating high FAPI uptake in the liver. In addition, the conditions with chronic inflammation or infection in the lung, such as bronchiectasis, rheumatological disorders with pulmonary manifestations, restrictive lung disease or infections might represent, among others, a limiting factor for pulmonary tumor diagnostic.

Most of our patient cohort showed a moderate uptake in the rectum without any known underlying pathology. Zheng et al. reported of this phenomenon and postulated that mild fibrous tissue hyperplasia associated with varicose veins and the inflammation as a cause of increased FAP expression [18]. Undoubtedly, persistent straining, pressure, and microinjuries in the epithelium of the anal canal might cause a certain kind of the so-called never healing wound which, as we know, leads to a stronger FAP expression. Moreover, the increased glucose metabolism that is often detected by high FDG uptake also supports the hypothesis of chronic inflammatory changes of the epithelium in the anal canal. However, to our knowledge, there is no significant literature data supporting the correlation between fibrous tissue hyperplasia in varicose veins and FAP expression. Similarly, in 57 out of 155 patients, we identified moderate uptake in the esophagus, predominantly in the lower third of the esophagus. Gastroesophageal reflux disease (GERD) is one of the most predominant gastrointestinal diseases with a prevalence in the US between 18.1–27.8% and 8.8–25.9% in Europe (an even higher incidence is to be assumed due to an availability of over-the-counter anti-acidic medication) [39, 40]. Studies suggest that cell damage and inflammation caused by gastric fluid may trigger the wound healing process and subsequent immunomodulation, promoted by inflammatory cytokines and chemokine activation [41]. Inflammatory cytokines cause fibroblast to myofibroblast transition, leading to fibrosis [42].

Beyond the characterization of benign findings in this study, we elaborated on the equivocal findings and classified them into benign and malignant lesions according to their etiology, which was determined by analyzing and crosschecking the electronic medical records including available pathology reports or reports of other imaging modalities. To our knowledge, a reliable parameter, or a diagnostic criterion such as a cutoff value for FAPI uptake is still missing. Our aim was to identify a pattern of FAPI uptake in benign lesions that could reliably predict the accurate diagnosis of equivocal findings in the clinical setting and thereby largely prevent misdiagnosis and mismanagement. Although there was sporadically overlap in the tracer uptake between benign and malignant lesions in some patients, we detected statistically significant differences in SUV_max_, SUV_mean_, and LBR in accordance with previous studies.

We performed ROC analysis to evaluate the diagnostic performance of [^68^ Ga]-FAPI PET/CT by assessing the semiquantitative metabolic parameters (SUV_max_, SUV_mean_, and LBR) in differentiation of equivocal findings on [^68^ Ga]-FAPI PET/CT, which yielded an excellent trade-off between sensitivity and specificity. We found a sensitivity of 78.8% and specificity of 85,8% at a SUV_max_ cutoff value of 5.5 under AUC of 0.89. Our evaluation showed better diagnostic accuracy at a SUV_mean_ cutoff value of 3.3 with a sensitivity of 84.9% and a specificity of 85,3% under AUC of 0.916. The cumulative accuracy and negative predictive factor for all three parameters are 82,8% and 89,7% respectively. Furthermore, the comparison of the ROC curves yielded a favorable result for the SUV_mean_, which, however, can be regarded as equivalent to other parameters. However, since the SUV_max_ and SUV_mean_ are not always comparable between different PET centers without cross-calibration of scanner hardware and reconstruction algorithms, the LBR might be a more reproducible predictive factor for the clinical and research setting as well. Since the FAP ligands, FAPI-02 and FAPI-04 are considered interchangeable due to comparable biodistribution at 1 h p.i. and similar diagnostic performance, no relevant influence on our analysis is expected [1].

Thus, our findings suggest rather promising, accurate diagnostic criteria for [^68^ Ga]-FAPI PET/CT distinguishing equivocal lesions based on semiquantitative metabolic parameters. We suggest that our findings can facilitate diagnostics by establishing predictable patterns of FAPI accumulation, especially while taking patient history into account. We have solely used semiquantitative parameters for the assessment, but if qualitative criteria such as the pattern and location of lesions, which can be evaluated only by a nuclear medicine physician, are also used, the diagnostic accuracy will probably increase further.

This investigation contained several limitations including its retrospective study design, with the associated risks of selection bias, since the cohort only consistsed of patients with known malignancies, frequently at advanced stages. Furthermore, the study is lacking a gold standard test for the validation of benign lesions. On the other hand, pathological analysis of every equivocal finding on [^68^ Ga]-FAPI PET/CT would be unfeasible and unethical. Finally, our results were obtained from a single institution and further multicenter studies are warranted to validate the utility of these metabolic threshold or cutoff values for differentiation of equivocal findings on [^68^ Ga]-FAPI PET/CT.

## Conclusions

Since the implementation of [^68^ Ga]-FAPI PET/CT imaging, benign processes with significant FAPI uptake challenge accurate and precise distinction for clinical and research purposes. Within this investigation, we established that benign pathophysiological changes overall show a significantly lower uptake than malignancies, implying that the fibroblast activation in both settings may have distinct underlying pathophysiological mechanisms. Furthermore, we aimed to predict semiquantitative metabolic cutoff values with SUV_max_, SUV_mean_, and LBR to propose a differentiation of benign and malignant findings in a representative patient cohort with various malignancies.

## Supplementary Information

Below is the link to the electronic supplementary material.

Supplementary file1 (DOCX 15 KB)

## Data Availability

The data used and/or analyzed during the current study are available from the corresponding author on reasonable request.

## References

[1] Loktev A, Lindner T, Mier W (2018). A tumor-imaging method targeting cancer-associated fibroblasts. J Nucl Med.

[2] Giesel FL, Kratochwil C, Schlittenhardt J et al. (2021) Head-to-head intra-individual comparison of biodistribution and tumor uptake of 68Ga-FAPI and 18F-FDG PET/CT in cancer patients. Eur J Nucl Med Mol Imaging. 10.1007/s00259-021-05307-1 10.1007/s00259-021-05307-1 PMC8566651 34137945

[3] Deng M, Chen Y, Cai L (2021). Comparison of 68Ga-FAPI and 18F-FDG PET/CT in the imaging of pancreatic cancer with liver metastases. Clin Nucl Med.

[4] Hamson EJ, Keane FM, Tholen S (2014). Understanding fibroblast activation protein (FAP): substrates, activities, expression and targeting for cancer therapy. Proteomics Clin Appl.

[5] Jansen K, Heirbaut L, Cheng JD (2013). Selective inhibitors of fibroblast activation protein (FAP) with a (4-quinolinoyl)-glycyl-2-cyanopyrrolidine scaffold. ACS Med Chem Lett.

[6] Cirri P, Chiarugi P (2011). Cancer associated fibroblasts: the dark side of the coin. Am J Cancer Res.

[7] Zi F, He J, He D (2015). Fibroblast activation protein α in tumor microenvironment: recent progression and implications (review). Mol Med Rep.

[8] Lindner T, Loktev A, Giesel F et al. (2019) Targeting of activated fibroblasts for imaging and therapy. EJNMMI Radiopharm Chem 4. 10.1186/s41181-019-0069-0 10.1186/s41181-019-0069-0 PMC6658625 31659499

[9] Rettig WJ, Garin-Chesa P, Beresford HR (1988). Cell-surface glycoproteins of human sarcomas: differential expression in normal and malignant tissues and cultured cells. Proc Natl Acad Sci U S A.

[10] Jacob M, Chang L, Puré E (2012). Fibroblast activation protein in remodeling tissues. Curr Mol Med.

[11] Bauer S, Jendro MC, Wadle A (2006). Fibroblast activation protein is expressed by rheumatoid myofibroblast-like synoviocytes. Arthritis Res Ther.

[12] Milner JM, Kevorkian L, Young DA (2006). Fibroblast activation protein alpha is expressed by chondrocytes following a pro-inflammatory stimulus and is elevated in osteoarthritis. Arthritis Res Ther.

[13] Brokopp CE, Schoenauer R, Richards P (2011). Fibroblast activation protein is induced by inflammation and degrades type I collagen in thin-cap fibroatheromata. Eur Heart J.

[14] Tillmanns J, Hoffmann D, Habbaba Y (2015). Fibroblast activation protein alpha expression identifies activated fibroblasts after myocardial infarction. J Mol Cell Cardiol.

[15] Schuberth PC, Hagedorn C, Jensen SM (2013). Treatment of malignant pleural mesothelioma by fibroblast activation protein-specific re-directed T cells. J Transl Med.

[16] Liu H, Yang X, Liu L (2021). Clinical significance of diffusely increased uptake of 68Ga-FAPI in thyroid gland. Front Med (Lausanne).

[17] Conen P, Pennetta F, Dendl K et al. (2022) 68 GaGa-FAPI uptake correlates with the state of chronic kidney disease. Eur J Nucl Med Mol Imaging. 10.1007/s00259-021-05660-1 10.1007/s00259-021-05660-1 PMC9308582 34988624

[18] Zheng S, Lin R, Chen S (2021). Characterization of the benign lesions with increased 68Ga-FAPI-04 uptake in PET/CT. Ann Nucl Med.

[19] Kratochwil C, Flechsig P, Lindner T (2019). 68Ga-FAPI PET/CT: tracer uptake in 28 different kinds of cancer. J Nucl Med.

[20] Giesel FL, Adeberg S, Syed M (2021). FAPI-74 PET/CT using either 18F-AlF or cold-kit 68Ga labeling: biodistribution, radiation dosimetry, and tumor delineation in lung cancer patients. J Nucl Med.

[21] Giesel FL, Kratochwil C, Lindner T (2019). 68Ga-FAPI PET/CT: biodistribution and preliminary dosimetry estimate of 2 DOTA-containing FAP-targeting agents in patients with various cancers. J Nucl Med.

[22] Dendl K, Finck R, Giesel FL (2021). FAP imaging in rare cancer entities-first clinical experience in a broad spectrum of malignancies. Eur J Nucl Med Mol Imaging.

[23] Dendl K, Koerber SA, Finck R et al. (2021) 68Ga-FAPI-PET/CT in patients with various gynecological malignancies. Eur J Nucl Med Mol Imaging. 10.1007/s00259-021-05378-0 10.1007/s00259-021-05378-0 PMC8484099 34050777

[24] Lindner T, Loktev A, Altmann A (2018). Development of quinoline-based theranostic ligands for the targeting of fibroblast activation protein. J Nucl Med.

[25] DeLong ER, DeLong DM, Clarke-Pearson DL (1988). Comparing the areas under two or more correlated receiver operating characteristic curves: a nonparametric approach. Biometrics.

[26] Mohmand-Borkowski A, Rozmyslowicz T (2021). Expression of fibroblast activation protein in human coronary vessels. Pol Merkur Lekarski.

[27] Puré E, Blomberg R (2018). Pro-tumorigenic roles of fibroblast activation protein in cancer: back to the basics. Oncogene.

[28] Fitzgerald AA, Weiner LM (2020). The role of fibroblast activation protein in health and malignancy. Cancer Metastasis Rev.

[29] Davidoff MS, Breucker H, Holstein AF (1990). Cellular architecture of the lamina propria of human seminiferous tubules. Cell Tissue Res.

[30] Meineke V, Frungieri MB, Jessberger B (2000). Human testicular mast cells contain tryptase: increased mast cell number and altered distribution in the testes of infertile men. Fertil Steril.

[31] Volpé R (1978). The pathology of thyroiditis. Hum Pathol.

[32] Smith TJ, Padovani-Claudio DA, Lu Y (2011). Fibroblasts expressing the thyrotropin receptor overarch thyroid and orbit in Graves’ disease. J Clin Endocrinol Metab.

[33] Hotta M, Sonni I, Benz MR (2021). 68Ga-FAPI-46 and 18F-FDG PET/CT in a patient with immune-related thyroiditis induced by immune checkpoint inhibitors. Eur J Nucl Med Mol Imaging.

[34] Krieger JN, Lee SWH, Jeon J (2008). Epidemiology of prostatitis. Int J Antimicrob Agents.

[35] Lim KB (2017). Epidemiology of clinical benign prostatic hyperplasia. Asian J Urol.

[36] Xu T, Zhao Y, Ding H (2021). 68GaGa-DOTA-FAPI-04 PET/CT imaging in a case of prostate cancer with shoulder arthritis. Eur J Nucl Med Mol Imaging.

[37] Levy MT, McCaughan GW, Abbott CA (1999). Fibroblast activation protein: a cell surface dipeptidyl peptidase and gelatinase expressed by stellate cells at the tissue remodelling interface in human cirrhosis. Hepatology.

[38] Levy MT, McCaughan GW, Marinos G (2002). Intrahepatic expression of the hepatic stellate cell marker fibroblast activation protein correlates with the degree of fibrosis in hepatitis C virus infection. Liver.

[39] Kellerman R, Kintanar T (2017). Gastroesophageal reflux disease. Prim Care.

[40] El-Serag HB, Sweet S, Winchester CC (2014). Update on the epidemiology of gastro-oesophageal reflux disease: a systematic review. Gut.

[41] Chiang CC, Chen C-M, Suen JL (2020). Stimulatory effect of gastroesophageal reflux disease (GERD) on pulmonary fibroblast differentiation. Dig Liver Dis.

[42] Kendall RT, Feghali-Bostwick CA (2014). Fibroblasts in fibrosis: novel roles and mediators. Front Pharmacol.

