# Supplementary material for: Distinguishing Benign and Malignant Findings on [68 Ga]-FAPI PET/CT Based on Quantitative SUV Measurements
Source: Mol Imaging Biol. 2022 Aug 23;25(2):324–33. doi: 10.1007/s11307-022-01759-5 (PMC10006041; doi:10.1007/s11307-022-01759-5)
Supplement: Supplementary file 1 — Supplementary file1 (DOCX 15 KB) [file 11307_2022_1759_MOESM1_ESM.docx]

**Supplement table 1:**

| **Site** | **University Hospital Heidelberg** |
| --- | --- |
| PET/CT scanner | Biograph mCT Flow, Siemens |
| Median injected activity (MBq), [^68^Ga]-FAPI | 239 (118 – 340) |
| CT reference (mAs) | 30 |
| CT peak kilovoltage (kV) | 130 |
| CT slice thickness (mm) | 5 |
| CT slice increment (mm) | 3 – 4 |
| PET reconstruction | OSEM algorithm |
| Iterations | 2 |
| Subsets | 21 |
| Matrix | 200 x 200 |
| Corrections | Gaussian FWHM 5.0 mm |
